# Parameter estimation of qualitative biological regulatory networks on high performance computing hardware

**DOI:** 10.1186/s12918-018-0670-y

**Published:** 2018-12-29

**Authors:** Muhammad Tariq Saeed, Jamil Ahmad, Jan Baumbach, Josch Pauling, Aamir Shafi, Rehan Zafar Paracha, Asad Hayat, Amjad Ali

**Affiliations:** 1 Research Centre for Modeling and Simulation (RCMS), NUST, Islamabad, 44000 Pakistan; 2 Chair of Experimental Bioinformatics, TUM School of Life Sciences Weihenstephan, Maximus-von-Imhof-Forum 3, Freising, 85354 Germany; 3 Computational Lipidomics group, Chair of Experimental Bioinformatics, TUM School of Life Sciences Weihenstephan, Maximus-von-Imhof-Forum 3, 85354, Freising, Germany; 4 Department of Computer Science, National University of Computer and Emerging Sciences, Lahore, Pakistan; 5 Atta-ur-Rahman School of Applied Bio sciences (ASAB), NUST, Islamabad, 44000 Pakistan; 6 UNIVERSITY OF MALAKAND, Chakdara, Khyber Pakhtunkhwa, 18000 Pakistan

**Keywords:** Biological regulatory networks, Qualitative modeling, Parameter estimation, René Thomas framework, Hexosamine biosynthetic pathway (HBP), Model checking, Parallel SMBioNet, MPJ express

## Abstract

**Background:**

Biological Regulatory Networks (BRNs) are responsible for developmental and maintenance related functions in organisms. These functions are implemented by the dynamics of BRNs and are sensitive to regulations enforced by specific activators and inhibitors. The logical modeling formalism by René Thomas incorporates this sensitivity with a set of logical parameters modulated by available regulators, varying with time. With the increase in complexity of BRNs in terms of number of entities and their interactions, the task of parameters estimation becomes computationally expensive with existing sequential SMBioNET tool. We extend the existing sequential implementation of SMBioNET by using a data decomposition approach using a Java messaging library called MPJ Express. The approach divides the parameters space into different regions and each region is then explored in parallel on High Performance Computing (HPC) hardware.

**Results:**

The performance of the parallel approach is evaluated on BRNs of different sizes, and experimental results on multicore and cluster computers showed almost linear speed-up. This parallel code can be executed on a wide range of concurrent hardware including laptops equipped with multicore processors, and specialized distributed memory computer systems. To demonstrate the application of parallel implementation, we selected a case study of Hexosamine Biosynthetic Pathway (HBP) in cancer progression to identify potential therapeutic targets against cancer. A set of logical parameters were computed for HBP model that directs the biological system to a state of recovery. Furthermore, the parameters also suggest a potential therapeutic intervention that restores homeostasis. Additionally, the performance of parallel application was also evaluated on a network (comprising of 23 entities) of Fibroblast Growth Factor Signalling in *Drosophila melanogaster*.

**Conclusions:**

Qualitative modeling framework is widely used for investigating dynamics of biological regulatory networks. However, computation of model parameters in qualitative modeling is computationally intensive. In this work, we presented results of our Java based parallel implementation that provides almost linear speed-up on both multicore and cluster platforms. The parallel implementation is available at https://psmbionet.github.io.

**Electronic supplementary material:**

The online version of this article (10.1186/s12918-018-0670-y) contains supplementary material, which is available to authorized users.

## Introduction

A long-standing goal of biomedical research is to identify potential therapeutic targets of complex human diseases (e.g. Cancer, HIV etc.) by employing system level modeling and analysis approaches. The study of bio-molecular interactions to understand genotype-phenotype relationships constitutes the core of Systems Biology [1]. The dynamics of biological regulations can be represented using variety of modeling frameworks [2]. These modeling approaches can be broadly categorized into continuous [3], discrete [4] and hybrid methods [5]. Continuous modeling approaches that use ordinary differential equations require precise parameter information, which in many cases cannot be extracted from noisy data obtained through experimental methods, such as microarrays, spectroscopy and biochemical kinetics. Qualitative modeling, on the other hand, inspired mainly from the work of Kauffman [6], and René Thomas [7, 8] uses a qualitative abstraction that allows to focus on logical connections between variables of a network rather than precise expression levels. Due to finite levels of expression, the degree of difficulty for parameter computation is less in qualitative modeling. The qualitative modeling framework [7, 8] can be used to capture important properties in biological networks such as stable steady states [9], bifurcation points, and cycles (homeostasis) [10]. These properties provide key insights into identification of the therapeutic targets [11] and further validation in wet labs. In order to model a BRN using this framework, values of logical parameters have to be provided. These model parameters are usually unknown and can be inferred using *formal method* technique such as *Model Checking* [12].

### Parameters estimation through model checking

Model Checking [13] is an automated technique for verification of complex hardware and software systems. Initially developed for concurrent program verification, model checking is now an industry standard methodology for proving correctness of digital circuits, security protocols and embedded systems. In many aspects, biological systems are similar to massively parallel software systems, characterized by non-deterministic behavior [14]. This analogy allows to use model checking for analysis of large number of possible outcomes of a biological model, similar to predicting behavior of a concurrent program.

Model checking approaches are differentiated on the basis of how they interpret the notion of time; Linear [15] or branching [12]. Due to branching nature of **C**omputation **T**ree **L**ogic (CTL), it is suitable to express properties of non deterministic dynamical systems such as BRNs, where a current state can have more than one successor states.

**Fig. 1:**
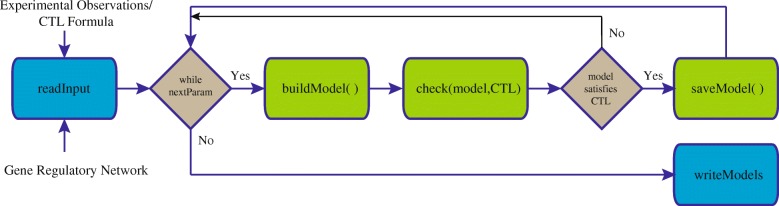
Flow of Parameter Estimation Approach using Model Checking. The sequential approach for parameter estimation exhaustively enumerates through all the possible combinations of logical parameters. For each parameter, it constructs a model which is evaluated against experimental observations by employing model checking. If the result of model checking is true, the model is appended in the list of selected models

Model Checking deciphers model parameters by using known observations about the expressions of the entities involved in a BRN [16, 17]. The sequential proceedure for estimation of logical parameters has been elucidated in Fig. 1. A Model checking tool takes a model $\mathcal {M}$ of BRN and its observations, formally expressed as property *ϕ* and then exhaustively explores $\mathcal {M}$ to verify *ϕ*. SMBioNet (Selection of Models of Biological Networks) [18] is an application which is based on qualitative framework, and uses NuSMV [19] as a model checker to find logical parameters of those models that satisfy known biological observations. However, due to large number of model parameters and its sequential implementation, this tool can be used for BRNs with number of genes less than 7 [20]. Modern High Performance Computing (HPC) platforms equipped with multicore processors and distributed memory clusters offer huge computational power to cope with the complexity of parameter inference for large BRNs.

Among the sequential algorithms that solve the problem of parameter inference by employing model checking, Bernot et al. [16] introduced this approach by finding model parameters for mucus production network in *Pseudomonas aeruginosa*. They implemented this approach in SMBioNet, which has been used for analysis of several BRNs, including biosurfactants production in *P. aeruginosa* [20], tail resorption network controlling metamorphosis in tadpole [18] and immunity control in bacteriophage lamda [21].

### Parallel parameter estimation methods

The use of parallel computing techniques to reduce complexity of biological systems has recently gained wide interest [22]. Barnat et al. [23] introduced an algorithm for partitioning of parameter estimation through Linear Temporal Logic (LTL) based parallel model checking. They defined the notion of *Parametrized kripke Structure* (PKS) to represent entire state space of model and parameters as single object, which is explored by multiple threads concurrently. On multicore platform with 8-cores, their parallel implementation achieved up to 6x speedup on regulatory networks of G1/S Cell Cycle Transition [24] and Ammonium Transport in E. Coli [25] using parallel LTL model checker [26].

Klarner et al. [27] introduced a technique for parameter identification by using LTL based colored model checking from behavioral properties and time series data [28]. The distribution of parameter space lead to roughly linear speed-up on 8-core platform on models of bacteriophage *λ* [29] and mammalian cell cycle [30]. A parallel approach to accelerate sequential algorithm was presented in [31] with preliminary results obtained through a wrapper implementation in C on top of existing Java implementation of SMBioNet [18].

The previous work has made significant contributions for development of LTL based efficient parameter estimation techniques [32–34]. On the other hand, it is established that for biological systems, which are non-deterministic, CTL is more suitable due to its branching nature [16, 35] and structured patterns for querying biological pathways [36]. Due to inherently sequential nature of CTL algorithm [37], it can not scale on modern multi-core and Cluster platforms. In this study, we present results of our parallel implementation that exploits application level parallelism in order to accelerate parameter estimation in qualitative modeling of biological networks. We employ a data parallel decomposition scheme that provides almost linear speed-up on multicore and cluster platforms. We extend the existing implementation of SMBioNet using the MPJ Express software, which is capable of executing on shared and distributed memory HPC hardware [1]. The details of the parallel implementation are accessible online at https://psmbionet.github.io.

## Methods

### Qualitative modeling framework

In this section, we briefly revisit the formal framework introduced by René Thomas as originally found in literature [38, 39].

#### **Definition 1**

(Biological Regulatory Network) “A Biological Regulatory Network (BRN) is a labeled directed graph *G*=(*V*,*E*), where *V* is a finite set of vertices, also called biological entities and *E*⊆*V*×*V* is the set of interactions.

The successors and predecessors of a biological entity are represented as $G_{\nu _{i}}^{+}$ and $G_{\nu _{i}}^{-}$, respectively. Each vertex is provided with a limit $\ell _{\nu _{i}} = \left |G_{\nu _{i}}^{+}\right |$ when $\left |G_{\nu _{i}}^{+}\right | \geq 1$, and $\ell _{\nu _{i}} = 1$ when $\left |G_{\nu _{i}}^{+}\right | = 0$. The edges are labeled by a pair (*τ*,*σ*), where $\tau \leq \ell _{\nu _{x}}$ is the threshold of influence, and *σ*={+,−} is called sign of interaction (+ for activation and - for inhibition). Each entity *ν*_*i*_∈*V* has its abstract expression level in the set $ E_{\nu _{i}} \,=\, \left \{0,1,....,r_{\nu _{i}}\right \}$ where $ r_{\nu _{i}} \!\leq \!l_{\nu _{i}}$. The *state* of a *BRN* is a configuration of expression levels of all biological entities at a particular time instant.

#### **Definition 2**

(State) A State of BRN is n-tuple $S = \left \{s_{\nu _{1}},.., s_{\nu _{n}}\right \}$, $\forall s_{\nu _{i}} \in E_{\nu _{i}}$, where $s_{\nu _{i}}$ is the abstract expression level of *ν*_*i*_.

The state space of a *BRN* is a cartesian product obtained on the range of expression levels of all entities and can be computed using Formula 1.

1 $$ \prod\limits_{i=1}^{n} E_{\nu_{i}}   $$

In a given state, each biological entity *ν*_*i*_ is regulated by its predecessors $G_{\nu }^{-}$, formally denoted as set of *resources*, $W_{\nu _{i}}$, defined as follows;

#### **Definition 3**

(Resources) Let *G*=(*V*,*E*) be a BRN. The set of resources $W_{\nu _{y}}$ of a variable *ν*_*y*_∈*V*, at level $s_{\nu _{y}}$, is defined as; $W_{\nu _{y}} = \left \{ \nu _{x} \in G^{-}_{\nu _{y}} | \left (s_{\nu _{x}} \geq \tau _{\nu _{x},\nu _{y}}\ and\ \alpha _{\nu _{x},\nu _{y}}=+\right) or \left (s_{\nu _{x}}< \tau _{\nu _{x},\nu _{y}}\ and \ \alpha _{\nu _{x},\nu _{y}}=-\right) \right \}$

In order to determine *resources* of an entity *ν*_*i*_, the presence of activators and absence of inhibitors is considered as *resource*. Consequently, $W_{\nu _{i}}$ contains inhibitors and activators of *ν*_*i*_. The targets towards which the levels of variables *ν*_*i*_ evolve, depend on the set of positive integers $K_{\nu _{i}}\left (W_{\nu _{i}}\right)$, also called *logical parameters*, indexed by $W_{\nu _{i}}$. The evolution operator (△) in the following formula shows next state towards which *ν*_*i*_ evolves.

2 $$ s_{\nu_{i}}\bigtriangleup K_{\nu_{i}}\left(W_{\nu_{i}}\right)= \left\{\begin{array}{lll} s_{\nu_{i}} + 1 & \texttt{if} & s_{\nu_{i}}< K_{\nu_{i}}\left(W_{\nu_{i}}\right)\\ s_{\nu_{i}} - 1 & \texttt{if} & s_{\nu_{i}} > K_{\nu_{i}}\left(W_{\nu_{i}}\right) \\ s_{\nu_{i}} & \texttt{if} & s_{\nu_{i}} = K_{\nu_{i}}\left(W_{\nu_{i}}\right) \end{array}\right.   $$

When variable *ν*_*i*_ has a certain expression level $s_{\nu _{i}}$, its evolution has three possibilities: (1) When $ s_{\nu _{i}} < K_{\nu _{i}}\left (W_{\nu _{i}}\right)$, the value of $s_{\nu _{i}}$ is incremented by one unit. Conversely, if $s_{\nu _{i}} > K_{\nu _{i}}\left (W_{\nu _{i}}\right)$, $s_{\nu _{i}}$ is decremented by one unit. However, $s_{\nu _{i}}$ does not evolve and remains constant, if $s_{\nu _{i}} = K_{\nu _{i}}\left (W_{\nu _{i}}\right)$.

The number of possible parameter combinations (*parametrization*), even for a small network, can be huge. Let *G*=(*V*,*E*) be a *BRN* with n*variables*, and |*G*^−^(*v*_*i*_)| be the cardinality of the regulators of *ν*_*i*_∈*G*, then number of possible *parametrization* can be computed using formula 3.

3 $$ \prod\limits_{i=1}^{n} \left(\ell_{v_{i}}+1\right)^{2^{\left|G^{-}\left(v_{i}\right)\right|}}   $$

#### **Definition 4**

(State Graph) Let *G* be a BRN and $s_{\nu _{a}}$ denotes the expression level of biological entity *a* in a state *s*∈*S*. Then the state graph *R*=(*S*,*T*) of *G*=(*V*,*E*) is a directed graph, where *S* represents set of states, and *T*⊆*S*×*S* is a relation between states, also called the transition relation, such that *s*→*s*^′^∈*T* iff:

- ∃ a unique *x* *ε* *V* such that $s_{\nu _{x}} \neq s_{\nu _{x}}' $ and $ s_{\nu _{x}}' = s_{\nu _{x}} \bigtriangleup K_{x}\left (W_{\nu _{x}}\right)$, and
- ∀$y ~ \epsilon ~\mathcal {V} \setminus \{x\} ~ s_{\nu _{y}}' = s_{\nu _{y}}$.” [38, 39].

Different updating schemes have been proposed for qualitative modeling of biological regulatory networks. These updating methods follow synchronous or asynchronous schemes [40]. In a synchronous qualitative model, all variables in the network evolve simultaneously with time. The synchronous mechanism is considered as computationally less expensive [40]. However, it is also less accurate because biological systems are considered asynchronous where changes in expression level of genes or proteins are not concurrent and take place at different time points [16, 21]. In this work, we use asynchronous updating scheme for construction of state graph. The asynchronous scheme is computationally expensive and therefore, we use parallel computing to reduce processing time [40].

**Fig. 2:**
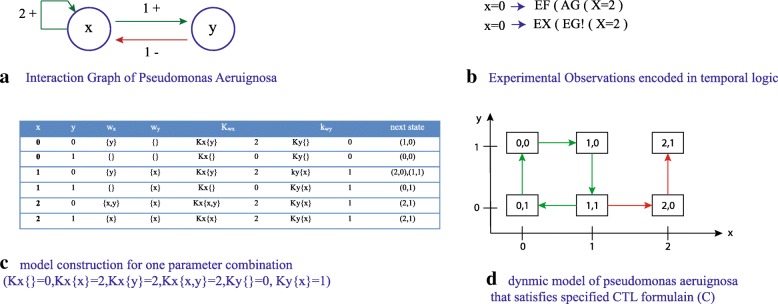
Qualitative Modeling Framework applied on a simple BRN. **a** BRN of *Pseudomonas aeruginosa* [16] shown as weighted directed graph by using Definition 1. The nodes/vertices represent genes whereas edges represent activation(s) and inhibition(s). The network comprises of two entities: x represents ALGU and y represents its inhibitor. **b** Experimental observations encoded as CTL (Computation Tree Logic) formula by using Definition 5. The formula describes a behavior in which the system exhibits normal and pathogenic responses (over-expression of gene X) in a single model that encodes two wet-lab observations is used for finding model parameters. **c**-**a** combination of logical parameters that satisfy CTL observations. **d** The dynamic model of the BRN is shown as a *State Graph* (see Definition 4). The state graph shows two important behaviors i.e. oscillation (homeostasis) as a cycle and a stable state (2,1) that represents a pathogenic behavior

To explain working of asynchronous qualitative modeling framework, we apply qualitative framework on BRN of *pseudomonas aeruginosa*. It is an opportunistic pathogen, commonly found in environment and responsible for mucus production in human lungs effected with *cystic fibrosis*. The BRN of *pseudomonas aeruginosa* is shown in Fig. 2a. It comprises of two entities i.e. ALGU (represented by node/vertex ’x’) and its inhibitor protein Anti-ALGU (represented by node/vertex ’y’). The activation and inhibition relationships are represented by using weighted directed edges by using Definition 1.

In order to measure parameters, experimental observations are encoded into temporal logic formulas. In case of *pseudomonas aeruginosa*, Fig. 2b shows two CTL formulas *ψ*_1_ and *ψ*_2_ that represent normal homeostasis and a pathogenic response receptively. In normal response, the expression level of gene x, starting from (*x*=0), does not reach (*x*=2). Whereas, when a pathogenic condition arises, the biological system reaches to a state where gene x is over-expressed finally leading to mucus production.

Figure 2c shows model construction for one parameter combination that satisfies experimental observations. For each state in biological system, its successor states are generated using Definitions 3 and 4. The model construction results in a dynamic model that provides useful insights such as stable steady states and deadlocks. Figure 2d shows the dynamic model as a *State Graph*(See Definition 4).

### Model checking

Model Checking is used to evaluate the dynamic model *M* against experimental observations expressed as formula CTL *ϕ*. The verification process determines correctness of *ϕ* in *M*, by employing a graph-theoretic procedure that exhaustively explores the entire state space of the system. Finally, model checker confirms correctness of *ϕ*, if formula is satisfied, or it produces a counter example to provide a trace of the execution path that violates *ϕ*. The counter example generation is a useful feature for diagnostic purposes.

In a CTL formula, We denote boolean true as ⊤ and boolean false as ⊥. The formula $(s_{\nu _{i}}=n)$ is true iff expression level of variable *ν*_*i*_, in current state, is equal to *n*. The CTL formula combines a set of connectives: ¬(negation), ∧ (logical AND), ∨ (logical OR) and ⇒ (implication) with temporal operators. The temporal operators are pairs of symbols; the first element of which is *A* (all paths) or *E* (at least one path), followed by *X* (next state), *F* (any future state) or *G* (all future states).

#### **Definition 5**

(CTL Formula) Let *G*=(*V*,*E*) be a BRN. A CTL formula *Φ* on *G* is defined as follows:

- atomic formulas are ⊤, ⊥ or any atomic proposition of the form (*ν*_*i*_=*n*), where *ν*_*i*_ is a variable in state graph and $n \in \left [0, \ell _{\nu _{i}}\right ]$.
- If *ϕ* and *ψ* are atomic formulas, then so are (¬*ϕ*), (*ϕ*∧*ψ*), (*ϕ*∨*ψ*), (*ϕ*⇒*ψ*), *Xϕ*, *EXϕ*, *AGϕ*, *EGϕ*, *EFϕ*, *AFϕ*, $(A\phi \bigcup \psi)$ and $(E\phi \bigcup \psi)$

### Parallel implementation in MPJ express

In general, parallel computations are divided into two categories based on communication requirements during the computation phase. The applications that do not need any communication during different computation phases are known as *embarrassingly parallel* computations. On the other hand, the applications that require frequent communication in between different computation phases are generally known as *synchronous* computations. One programming approach to implement the embarrassingly parallel computations is to use the “master/slave” model. Since the parameter estimation problem that we are tackling in this study is embarrassingly parallel in nature, we employ the master/slave model to produce the parallel code [1].

Embarrassingly parallel applications are parallelized using master/slave model typically involving three stages. In the first stage, the master process reads the input data, performs domain decomposition, and communicates the relevant chunk to each slave process. The second stage is the computation stage, where all worker processes perform parameter estimation on their own data. During the third and the final stage, all slave processes communicate results back to the master process that generates output for the end user. Out of all three stages, the second stage i.e. the computation phase typically requires the most processing time. In embarrassingly parallel applications, there is no communication required during computation phase, leading to almost linear speedup [1].

#### Problem decomposition

Here, we use two approaches for parameter decomposition [1].

- The first approach exploits the data parallel nature of parameter estimation problem [31]. The parameter state space is partitioned among available processors. We refer to this as coarse-grained parallelism that employs high level data parallelism.
- The second approach harnesses fine-grained parallelism available in parallel model checkers [26, 41–43] where the underlying algorithms partitions a state graph for verification of biological behaviors encoded in temporal logic.

#### Coarse grained parallelism

The first partitioning scheme we use in our study divides parameter space into mutually exclusive regions—explored by different worker processes. Since a new model needs to be constructed for each parameter combination, these regions can be explored in parallel by a collection of processes referred to as processing elements (PE). Each PE inspects only a subset of parameter space; and for each combination in that space, a state graph/model is generated. The verification is performed by invoking model checker as an external process to determine whether CTL observations are true. Finally, a reduction operation involves a communication step for receiving accepted sets of parameters. Treating each valuation of parameter separately allows to formulate task of parameter estimation as high level data parallel problem and the decomposition is embarrassingly parallel without any significant communication.

In this study, we use master/worker model of computation to implement high level data parallelism. An important step in parallelizing the code is to perform domain decomposition or partitioning of the input data at the master process. We employ primitive block domain decomposition to produce equally-sized independent chunks of input data for each worker process.

**Fig. 3:**
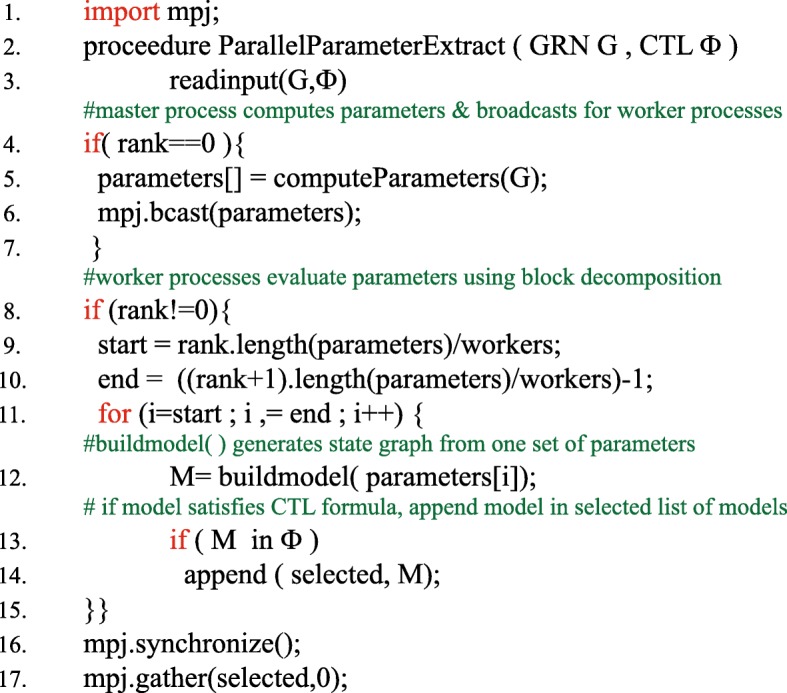
Pseudo-code of parallel implementation employing coarse-grained parallelism

We implement our partitioning strategy using the current implementation of SMBioNet. Figure 3 shows pseudocode that uses coarse grained decomposition. The *for* loop (line 11) in Fig. 3 shows that each worker process performs a block decomposition in order to identify a subset of the total parameter space it needs to explore. For each combination in that space, a new model is generated and provided to symbolic model checker NuSMV [19, 44] to determine correctness of CTL properties. If model checker satisfies the formula, the parameter estimation algorithm appends the model in the list of selected models.

Once the computation phase is over, the reduction step is required to write selected model parameters in a single output file. At this point, each worker process sends its list of selected parameters to the master process that receives selected parameters and produces a single output file. The complexity of the communication involved in reduction step is linear in terms of number of models satisfying the CTL property.

#### Fine grained parallel decomposition

Although a high-level decomposition scheme provides a good partitioning strategy for distributed memory architectures (HPC Clusters) due to low communication cost, a lower level decomposition of parameter estimation is motivated by increasing computational capabilities of shared memory multicore computers. Moreover, the maximum speedup achieved by high-level data parallelism is bounded by serial factor to evaluate one set of parameters. In theory, the integration of a multi-threaded model checker implementation such as Java Temporal Logic Framework (JTLV) [45] at multicore level can further reduce processing time. But the potential speedup depends on the granularity of tasks. In practice, the parameter estimation problem comprises of large number of small ’work units’ such that the complexity of each task is *O*(|*S*|.*ψ*). The symbolic model checking algorithm uses Binary Decision Diagrams (BDDs) as internal data structure for state representation. The multi-threaded packages such as JTLV do not parallelize the core operations involved in BDD computation. In turn, a task-parallel simulation using JTLV can provide relatively better performance when several CTL formulas are to be verified independently. One such case is the use of temporal logic patterns to check all the potential qualitative states for a specific property [36]. The related work on parallelization of core BDD operations for multicore processors is notably absent [46]. van Dijk and van de Pol [47] introduce a BDD package Sylvan that demonstrates a non-linear speedup up to 12X on large models.

## Results and discussion

In order to validate results of our parallel implementation, we undertake case study of Hexosamine Biosynthetic Pathway (HBP) and its involvement in Cancer [1, 48].

Additionally, for performance evaluation we use three models of biological pathways selected from the literature. These include tail resorption network during tadpole metamorphosis [18], immunity control in lambda phage [29, 35], MAL-associated pathway controlling Cerebral Malaria [39] and qualitative model of Fibroblast Growth Factor (FGF) Signalling in *Drosophila melanogaster* [49].

### Case study 1: role of O-linked N-acetylglucosamine transferase (OGT) in cancer

Cancer caused by complex genetic alterations is a diverse group of diseases. The amplification of oncogenes such as MYC, PI3K and EGFR and down regulation of tumor suppressor proteins is well established. There is a growing evidence about glycolytic fueling of cancer cells that results in oncogenic activation, evasion of apoptosis and proliferation of cancer cells. Fardini et al. [50] proposed O-GlcNAcylation as a new hallmark and approach for treatment of cancer. Increased expression of OGT has been reported in various types of cancer, including cancer of breasts, lungs, liver, bladder, endometrium, prostate, pancreas, and colon [51–57].

**Fig. 4:**
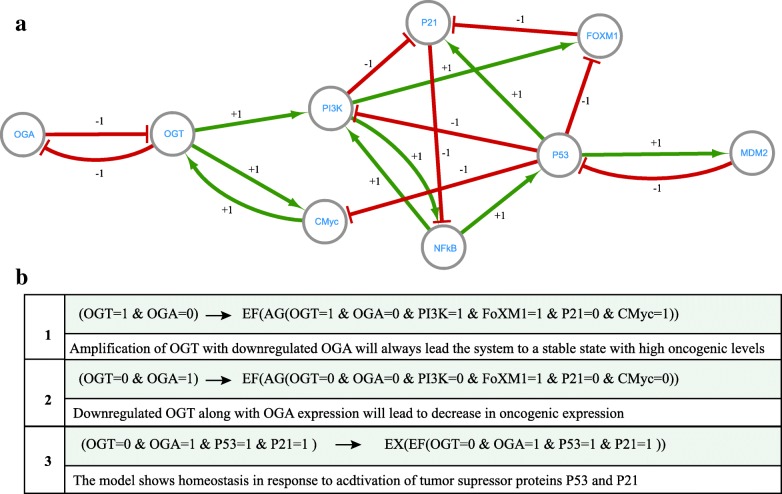
**a** Qualitative Biological Regulatory Network (BRN) of Hexosamine Biosynthetic Pathway (HBP) intersection with PI3K-mTOR-Myc signaling and P53-MDM2 signalling axis. **b** CTL observations used in [48] to generate dynamic model in the form of stategraph (as shown in Fig. 5b. **a** HBP intersection is shown with PI3K-mTOR-Myc siganling nd P53-MDM2 signalling axis. The increased flux of HBP is responsible for hyper O-GlcNAcylation which is implicated in several types of cancers. HBP generates UDP-GlcNAC (Urdine diphosphate N-acetylglucosamine) which is consumed by OGT. Hyper O-GlcNAcylation of CMyc triggers PI3K-mTOR-MYC signalling axis which is involved in cross talk with Forkhead box M1 (FoxM1). FoxM1 is further regulated by OGT. The qualitative BRN shows interconnections of important entities. The nodes/circles represent biological entities whereas interactions between two entities are represented with arrows. There are two types of interactions: activations (labelled with pointed green arrows) and inhibitions (labelled with blunt red arrows). The weight of the arrows indicate threshold of interaction. (see Definition 1). **b** Three CTL observations used in [48] are listed. These CTL observations are used by parallel SMBioNet implementation for estimation of parameters

**Fig. 5:**
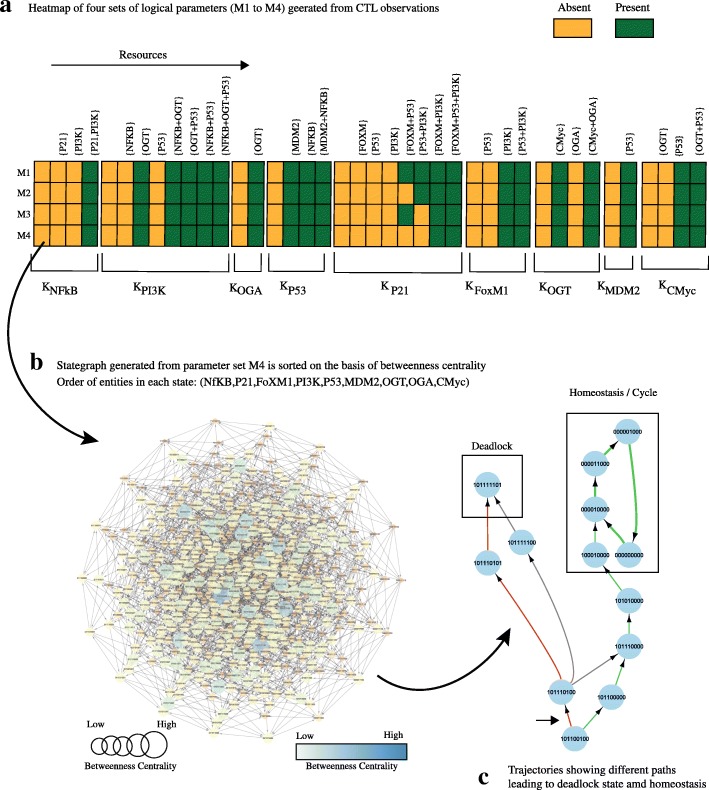
Role of OGT in Cancer Progression: Parameter Estimation, qualitative model generation and discovery of important properties(A-C). In (**a**) Heatmap of four sets of logical parameters is shown. These parameters are computed from CTL observations used in [48] by using our Parallel Implementation. The parameter sets are numbered from M1 to M4. **b** The stategraph is generated from parameter set M4 used in [48].The stategraph comprises of 512 nodes and 2304 edges. The order of states in stategraph is (NFkB,P21,FoXM1,PI3K,P53,MDM2,OGT,OGA,Cmyc). (**c**) The trajectories in the qualitative model show different possibilities reaching to the deadlock state (1,0,1,1,1,1,1,0,1) with high oncogenic expression levels along with elevated OGT levels or homeostasis/cycle comprising of four states

A qualitative model of the Hexosamine Biosynthetic pathway (HBP) explaining the association between hyper O-GlcNAcylation and cancer progression was developed by [48]. The qualitative BRN (see Fig. 4a comprised of 9 entities and three CTL observations for parameters computation. (See Fig. 4). The first CTL observation searches for a stable state with high expression of oncogenes. When a dynamic model, in the form of a state-graph is generated (Fig. 5b, it shows a deadlock state (1,0,1,1,1,1,1,0,1) along with normal homeostasis of P53-MDM2 oscillations (Fig. 5c. From a qualitative state (1,0,1,1,0,0,1,0,0), the biological system can follow different trajectories, leading to the deadlock state (1,0,1,1,1,1,1,0,1) or normal homeostatic behavior (cycle). The exact course of a biological system’s progression towards a target depends on the order of successive alterations in gene expressions. For example, sustained activation of OGT along with positive feedback from CMyc results in a deadlock state. Once the biological system reaches a deadlock state, it cannot recover to normal homeostatic response or to another qualitative state.

**Fig. 6:**
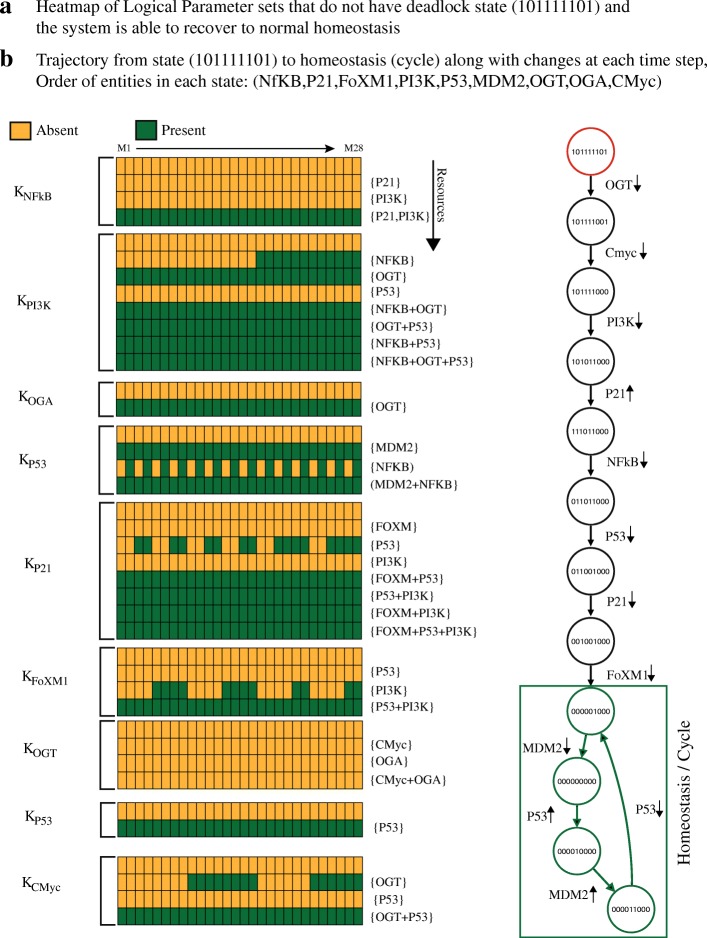
**a** Parameter estimation after therapeutic intervention. **b** Trajectory showing recovery from high oncogenic levels to normal homeostasis. We eliminate of CTL observation (OGT=1,OGA=0) → EF(AG(OGT=1,OGA=0,PI3K=1,FoXM1=1,P21=0,CMyc=1)) that encodes the occurrence of the deadlock state with high oncogenic expression levels. The parameter computation results show 28 different sets of logical parameters. These models do not have the deadlock state (1,0,1,1,1,1,1,0,1) reported in [48]. The parameter estimation was performed using our parallel implementation. The parameters are numbered from M1 to M2 and rendered as a heatmap. Each column represents a unique set of parameters. The expression of parameters is represented with green rectangles whereas the down-regulated values are shown as orange rectangles. The result shows that in order to avoid the deadlock state, the parameters of OGT must be at low expression. **b** The trajectory in the qualitative model obtained from one of the 28 parameter sets show that from state (1,0,1,1,1,1,1,0,1), the system is able to reach to normal homeostasis (cycle). The order of states is (NFkB,P21,FoXM1,PI3K,P53,MDM2,OGT,OGA,Cmyc)

In order to suggest a potential therapeutic target that forces the biological system to move from the deadlock state to homeostasis, it is important to compute logical parameters for which the dynamic models do not have qualitative state (1,0,1,1,1,1,1,0,1) as the deadlock state. The computation of these parameters form the basis of any therapeutic intervention to restore homeostasis. Therefore, we modified CTL observations used in [48] by eliminating the first CTL property (Fig. 4b. The source code of input models used in this study is available as Additional file 1. The new parameter configurations were computed using our parallel implementation. As a result, 28 parameter sets computed using modified CTL are rendered as heatmap in Fig. 6a. The heat-map suggests four critical resources of OGT: {} denoting the absence of CMyc (activator) and presence of OGA(inhibitor), {*CMyc*} denoting presence of CMyc and OGA, {*OGA*} denoting absence of OGA and CMyc and {*CMyc,OGA*} showing presence of CMyc and absence of OGA (see Definition 3).

The main difference between the two sets of parameters: the ones having the deadlock state (1,0,1,1,1,1,1,0,1) and those shown in Fig. 6 is that in the later sets, OGT-CMyc loop is down-regulated. Since CMyc is the activator of OGT, it should also be kept at a low expression level along with OGT. Thus, the results suggest an integrative therapeutic strategy for the treatment of cancer. The qualitative trajectories in the modified model show that the sustained down-regulation of these two genes results in restoration of P53-MDM2 oscillations and recovery to normal homeostasis. One such trajectory is shown in Fig. 6b. As the biological system progresses from one qualitative state to another, the changes in gene expression at each step is highlighted in Fig. 6b. The simulation results show that under the influence of computed logical parameters, the biological system moves from the qualitative state (1,0,1,1,1,1,1,0,1) to a cycle comprising of four states: (0,0,0,0,0,1,0,0,0), (0,0,0,0,0,0,0,0,0), (0,0,0,0,1,0,0,0,0) and (0,0,0,0,1,1,0,0,0). This cycle serves as an important attractor for the normal homeostatic response to take over.

### Case study 2: parameters scanning of the qualitative model of fibroblast growth factor (FGF) signalling in *drosophila melanogaster*

**Fig. 7:**
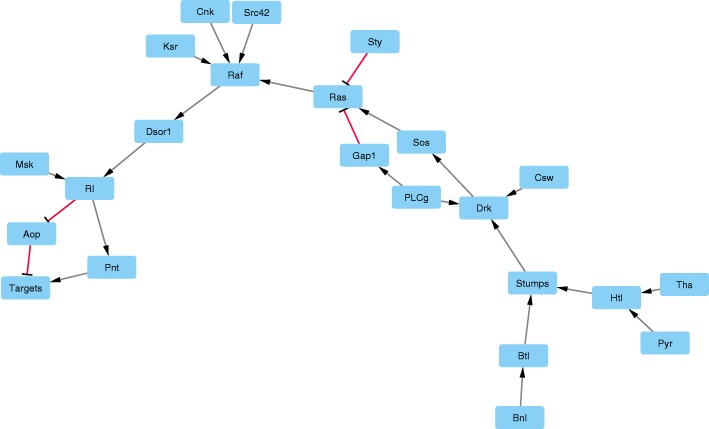
Qualitative model of Fibroblast Growth Factor (FGF) signalling in *Drosophila melanogaster*, adapted from [49] and rendered by using Cytoscape software [62]

Additionally, a large model of Fibroblast Growth Factor (FGF) Signalling in *Drosophila melanogaster*, comprising of 23 genes is considered as a benchmark to demonstrate the use of HPC. We calculated parameters for an important CTL property that leads to a stable state in FGF model (Additional file 2). *Drosophila Melanogaster*, a specie of fly belongs to the family of *Drosophilidae* and is known generally as fruit fly or vinegar fly. It has been used as a model to study cellular signaling involving growth factors that may have played a role in transition from single cells to more sophisticated multi-cellular organisms [58, 59]. The role of growth factors regulation in cellular differentiation towards the evolution of multicellular organisms is a well studied topic in systems biology. The recent research work carried out indicates that Fibroblast Growth Factor (FGF) signaling plays an important role in inducing changes in cellular behavior. The involvement of FGF in controlling cellular behavior in mammals was first identified with the discovery of FGF receptor in *Drosophila melanogaster*. The low genetic redundancy of *Drosophila* makes it an attractive model system to study FGF signaling. Thieffry et al. constructed various logical models of different pathways implicated in *Drosophila* signaling [49]. We used logical model constructed by Thieffry et al. [49] (available in GINsim database [60]) to evaluate the performance of our parallel approach. The logical model of FGF signaling in *Drosophila* comprises of 23 entities it is shown in Fig. 7. The state space of the model comprises of 8.3 ×10^6^ qualitative states. The SMBioNet code of the model is provided in Additional file 2.

### Performance evaluation

**Fig. 8:**
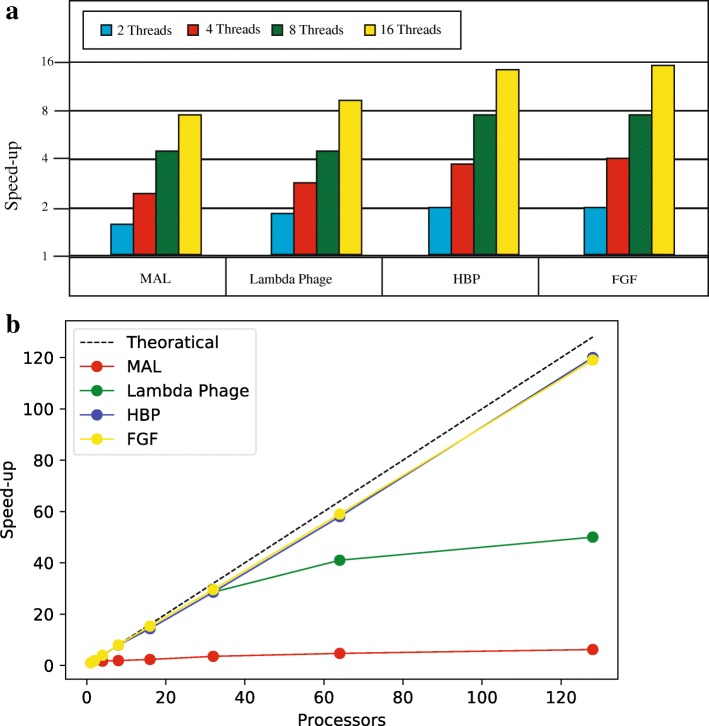
Speed-up observed on four different models in multicore (**a**) and cluster mode (**b**): The experimental results show almost linear speedup for parameter estimation of Hexosamine Biosynthetic Pathway. The observed speed-up decreases with decrease in the size of model due to reduced granularity

We carried out performance benchmarking on aforementioned networks. The running time (in seconds) and observed speedup in multicore and cluster mode are shown in Fig. 8. When executing our parallel code in the multicore mode, we employed a Dual Quad Core Intel Xeon PC (2.24GHz), equipped with 24GB of memory. We also enabled Hyper-Threading that allowed us to launch 16 threads using MPJ Express on this platform. The execution time for tail resorption network, 2, 4, 8 and 16 threads is plotted in Fig. 8a. In the multicore mode, the parallel Java application executes on a single system comprising of shared memory or multicore processors. Internally the MPJ Express now executes a single OS process with multiple threads [61] to harness the computational power offered by multicore systems.

The running time (in seconds) and speedup achieved on four input models is shown Fig. 8. The observed speedup shows that scalability improves with increase in the size of input models. For smaller models, the maximum speedup is not linear when number of threads are increased to maximum. This is due to two reasons: (1) small granularity of operations carried out by threads and (2) overhead of invoking a model checker as an external process. The performance in the multicore mode is better on tadpole’s tail resorption network. The observed speedup is almost linear. Generally, the coarse-grained decomposition can scale linearly due to low communication overhead in shared memory model.

In the cluster mode, we evaluated performance of our parallel approach on 32 node HPC Cluster, hosted at RCMS National University of Sciences and Technology, Pakistan. Each compute node was equipped with dual quadcore Intel Xeon E5520 processors with 24GB of RAM. The nodes are inter-connected via Gigabit Ethernet and QDR InfiniBand (40 Gbps). The software environment consisted of MPJ Express 0.40, Oracle JDK 1.7.0 25 version and GNU GCC 4.8.1. In the cluster mode, parallel applications execute in a typical cluster environment where processing elements are connected to one another using a fast interconnect like Infiniband and Myrinet. The MPJ Express software provides various communication devices for various interconnects.

One limitation of this approach is that individual processes are likely to suffer from state space explosion—a major limitation of the underlying exhaustive model checking algorithm. The state space of a BRN is a cartesian product obtained on the range of expression levels of all entities and given as a comma-separated string of ones and zeros. When the state graph is too large for single system’s memory, a high level data parallel approach will suffer from state space explosion. Each qualitative state in a BRN has a maximum of n outgoing transitions. The total number of state graphs for a boolean network with *n* genes is $2\left (n^{2^{n}}\right)$. By comparing the worst case complexities the two aforementioned approaches, we argue that parameter synthesis has more computational complexity than memory requirements, which paves the way for using high level data parallelism (HL-DP) on a distributed memory architecture. The distributed memory systems are exemplified by commodity compute clusters—group of computers connected using a fast and private network— where multiple processing elements communicate to one another using some form of messaging to solve a single problem. High level data parallelism for distributed memory systems is achieved by the Message Passing Interface MPI standard, which is considered as the de facto API for programming parallel applications. The most popular implementations of MPI include MPICH and Open MPI for C, and MPJ-Express for Java language. The main idea of our parallel implementation is based on partitioning of parameter space. Instead of using a parameterized Kripke structure, treating each valuation of parameter separately allows to formulate task of parameter estimation as data parallel problem and therefore achieves a more linear speed-up. The running time for tail resorption network, for 2,4,8,16,32,64,128 processes, is plotted in Fig. 8.

**Fig. 9:**
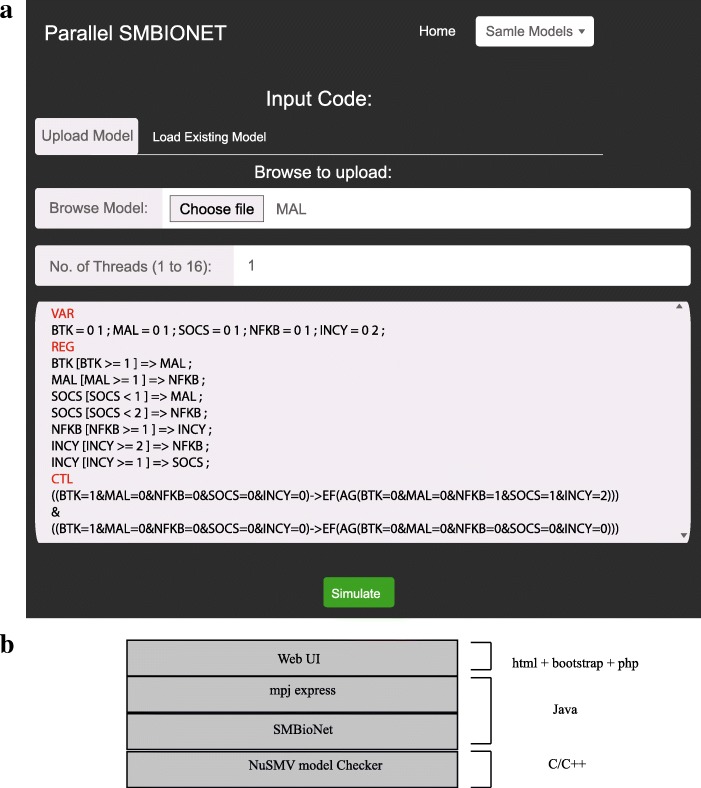
Web-based user interface (**a**) and architecture (**b**) of Parallel Implementation: The user can upload or select from a list of existing models by using the web based interface. The number of threads are specified before clicking the simulate button. The software calls the underlying mpj express engine to execute the simulation on specified number of threads. The model is specified as an input file which is divided into four section; VAR: for defining biological entities (genes, proteins etc.) and their thresholds, REG: for specifying interactions, PARA: for specifying range of logical parameters is an optional section, and CTL: for defining CTL formulas. The software comprises of four layers (as shown in **b**). The user interface layer is implemented by using web programming APIs (HTML, PHP and Bootstrap), MPJ Express is used for parallelization of the underlying sequential implementation which finally invokes NuSMV model checking for formal verification of model against CTL properties

The architecture of our parallel implementation is shown in Fig. 9b. It comprises of four layers. The existing implementation of SMBioNet is shown as an intermediate layer that is developed in Java. The only non-Java component is the NuSMV model checker, which is developed in C/C++ language but it is supported on Linux and Windows platform. In this way, our parallel implementation can can be installed on Windows and wide variety of Linux platforms. We made two important extensions that makeup the P-SMBioNet package; firstly, we add support for parallelization that enables our implementation to take advantage of raw computational power offered by modern multi-core and cluster computers. Secondly, we added a web based Graphical User Interface (GUI) for convenient model construction and state graph analysis from a remote system.

## Conclusion

Parameter inference is a key challenge in qualitative modeling of biological regulatory networks. Model checking techniques are used to decipher values of parameters from known biological observations expressed as temporal logic formulas. However, with increase in the size of network, complexity of parameter estimation algorithm increases exponentially. Therefore, efficient computational techniques are required to reduce the processing time of parameters computation. In this study, we investigated the use of parallel computing to accelerate parameter inference procedure by using a Java based library MPJ Express. We extended the sequential implementation by partitioning the parameter space and evaluated our parallel implementation on multicore and cluster platforms. We undertook a case study of the Hexosamine Biosynthetic Pathway (HBP) and its involvement in cancer progression. Through parameter computation with our parallel implementation, we were able to suggest a therapeutic intervention that can lead the system from a deadlock state to normal homeostasis. The experimental results obtained on a 23 genes network of Fibroblast Growth Factor in *Drosophila melanogaster* indicate that our approach is scalable and reduces execution time. Furthermore, our parallel implementation can be used through a web-based interface that can be accessed online. The reduction in execution time shows that this approach can be used in parameter inference applications on multicore desktop computers and laptops, and on special distributed architectures such as clusters. In future, we aim to provide a graphical editor for creating of qualitative models and construction of CTL properties. Moreover, we also aim to provide support for synchronous computation of the state graphs.

## Availability and requirements

**Project name:** Parallel SMBioNet**Project home page:**https://psmbionet.github.io**Operating system(s):** Linux, Tested with CentOS 6.5**Programming language:** Java**Other requirements:** SMBioNet, MPJExpress 0.41, NuSMV 2.4.3**License:** GPL

## Additional files

Additional file 1 SMBIONET FILE 1. The input file used for computing model parameters for the Hexosamine Biosynthetic Pathway (HBP). The sections entitled VAR, REG, PARA and CTL corresponds to the allowed expression levels of entities, interactions in the Biological Regulatory Network(BRN), permissible parameter range of each entity and CTL formulas, respectively. (ZIP 1 kb)

Additional file 2 SMBIONET FILE 2. The SMBioNet file contains source code of qualitative model of Fibroblast Growth Factor (FGF) Signalling in Drosophila melanogaster. (ZIP 1 kb)

## References

[1] Saeed MT. Formal modelling and analysis of the role of hexosamine biosynthetic pathway in cancer: Exploiting parallelism in qualitative biological regulatory networks. 2018. PhD thesis, Research Centre for Modelling and Simulation (RCMS), National University of Sciences and Technology (NUST), Islamabad, Pakistan.

[2] De Jong H (2002). Modeling and simulation of genetic regulatory systems: a literature review. J Comput Biol.

[3] Mestl T, Plahte E, Omholt SW (1995). A mathematical framework for describing and analysing gene regulatory networks. J Theor Biol.

[4] Albert R. Boolean modeling of genetic regulatory networks. In: Complex Networks. Springer: 2004. p. 459–81.

[5] Ahmad J, Bernot G, Comet J-P, Lime D, Roux O (2007). Hybrid modelling and dynamical analysis of gene regulatory networks with delays. ComPlexUs.

[6] Glass L, Kauffman SA (1973). The logical analysis of continuous, non-linear biochemical control networks. J Theor Biol.

[7] Thomas R (1978). Logical analysis of systems comprising feedback loops. J Theor Biol.

[8] Atkinson DE (1965). Biological feedback control at the molecular level. Science.

[9] Snoussi EH, Thomas R (1993). Logical identification of all steady states: the concept of feedback loop characteristic states. Bull Math Biol.

[10] Thomas R. On the relation between the logical structure of systems and their ability to generate multiple steady states or sustained oscillations. In: Numerical Methods in the Study of Critical Phenomena. Springer: 1981. p. 180–93.

[11] Materi W, Wishart DS (2007). Computational systems biology in drug discovery and development: methods and applications. Drug Discov Today.

[12] Clarke EM, Emerson EA. Design and Synthesis of Synchronization Skeletons Using Branching Time Temporal Logic: Springer; 1982.

[13] Clarke EM, Grumberg O, Peled D. Model Checking: MIT press; 1999.

[14] Fisher J, Henzinger TA (2007). Executable cell biology. Nat Biotechnol.

[15] Pnueli A. The temporal logic of programs. In: Foundations of Computer Science, 1977., 18th Annual Symposium On. IEEE: 1977. p. 46–57.

[16] Bernot G, Comet J-P, Richard A, Guespin J (2004). Application of formal methods to biological regulatory networks: extending thomas’ asynchronous logical approach with temporal logic. J Theor Biol.

[17] Carrillo M, Góngora PA, Rosenblueth DA (2012). An overview of existing modeling tools making use of model checking in the analysis of biochemical networks. Front Plant Sci.

[18] Khalis Z, Comet J-P, Richard A, Bernot G (2009). The smbionet method for discovering models of gene regulatory networks. Gene Genomes Genom.

[19] Cimatti A, Clarke E, Giunchiglia E, Giunchiglia F, Pistore M, Roveri M, Sebastiani R, Tacchella A. Nusmv 2: An opensource tool for symbolic model checking. In: Computer Aided Verification. Springer: 2002. p. 359–64.

[20] Richard A, Rossignol G, Comet J-P, Bernot G, Guespin-Michel J, Merieau A (2012). Boolean models of biosurfactants production in pseudomonas fluorescens. PloS ONE.

[21] Richard A, Comet J-P, Bernot G. Formal methods for modeling biological regulatory networks. In: Modern Formal Methods and Applications. Springer: 2006. p. 83–122.

[22] Ballarini P, Guido R, Mazza T, Prandi D (2009). Taming the complexity of biological pathways through parallel computing. Brief Bioinform.

[23] Barnat J, Brim L, Krejci A, Streck A, Safranek D, Vejnar M, Vejpustek T (2012). On parameter synthesis by parallel model checking. IEEE/ACM Trans Comput Biol Bioinforma (TCBB).

[24] Swat M, Kel A, Herzel H (2004). Bifurcation analysis of the regulatory modules of the mammalian g1/s transition. Bioinformatics.

[25] Ma H, Boogerd FC, Goryanin I (2009). Modelling nitrogen assimilation of escherichia coli at low ammonium concentration. J Biotechnol.

[26] Barnat J, Brim L, Ceska M, Rockai P. Divine: Parallel distributed model checker. In: Parallel and Distributed Methods in Verification, 2010 Ninth International Workshop On, and High Performance Computational Systems Biology, Second International Workshop On. IEEE: 2010. p. 4–7.

[27] Klarner H, Streck A, Šafránek D, Kolčák J, Siebert H. Parameter identification and model ranking of thomas networks. In: Computational Methods in Systems Biology. Springer: 2012. p. 207–26.

[28] Klarner H, Siebert H, Bockmayr A (2012). Time series dependent analysis of unparametrized thomas networks. IEEE/ACM Trans Comput Biol Bioinforma (TCBB).

[29] Thieffry D, Thomas R (1995). Dynamical behaviour of biological regulatory networks—ii. immunity control in bacteriophage lambda. Bull Math Biol.

[30] Fauré A, Naldi A, Chaouiya C, Thieffry D (2006). Dynamical analysis of a generic boolean model for the control of the mammalian cell cycle. Bioinformatics.

[31] Tariq Saeed JA. A parallel approach for accelerated parameter identification of gene regulatory networks. In: Proceedings of the 2nd International Work Conference on Bioinformatics and Biomedical Engineering (IWBBIO), 7-9 April 2014; Spain: 2014.

[32] Barnat J, Brim L, Ročkai P. Scalable multi-core ltl model-checking. In: Model Checking Software. Springer: 2007. p. 187–203.

[33] Laarman AW. Scalable Multi-core Model Checking: University of Twente; 2014.

[34] Barnat J, Bauch P, Brim L, Češka M (2012). Designing fast ltl model checking algorithms for many-core gpus. J Parallel Distrib Comput.

[35] Richard A, Comet J-p, Bernot G, Methods F. Formal Methods for Modeling Biological Regulatory Networks. 2014.

[36] Monteiro PT, Ropers D, Mateescu R, Freitas AT, de Jong H (2008). Temporal logic patterns for querying dynamic models of cellular interaction networks. Bioinformatics (Oxford, England).

[37] Beyersdorff O, Meier A, Thomas M, Vollmer H, Mundhenk M, Schneider T. Model checking ctl is almost always inherently sequential. In: Temporal Representation and Reasoning, 2009. TIME 2009. 16th International Symposium On. IEEE: 2009. p. 21–28.

[38] Bernot G, Cassez F, Comet J-P, Delaplace F, Müller C, Roux O (2007). Semantics of biological regulatory networks. Electron Notes Theor Comput Sci.

[39] Ahmad J, Niazi U, Mansoor S, Siddique U, Bibby J (2012). Formal modeling and analysis of the mal-associated biological regulatory network: Insight into cerebral malaria. PLoS ONE.

[40] Garg A, Di Cara A, Xenarios I, Mendoza L, De Micheli G (2008). Synchronous versus asynchronous modeling of gene regulatory networks. Bioinformatics.

[41] Barnat J, Brim L, Černá I, Dražan S, Šafránek D (2008). Parallel model checking large-scale genetic regulatory networks with divine. Electron Notes Theor Comput Sci.

[42] Holzmann GJ, Bosnacki D (2007). The design of a multicore extension of the spin model checker. Softw Eng IEEE Trans.

[43] Holzmann GJ, Bosnacki D. Multi-core model checking with spin. In: Parallel and Distributed Processing Symposium, 2007. IPDPS 2007. IEEE International. IEEE: 2007. p. 1–8.

[44] Chabrier N, Fages F. Symbolic model checking of biochemical networks. In: Computational Methods in Systems Biology. Springer: 2003. p. 149–162.

[45] Pnueli A, Sa’ar Y, Zuck LD. Jtlv: A framework for developing verification algorithms. In: CAV. Springer: 2010. p. 171–4.

[46] Van Dijk T, Laarman A, Van De Pol J (2013). Multi-core bdd operations for symbolic reachability. Electronic Notes in Theoretical Computer Science.

[47] van Dijk T, van de Pol J (2017). Sylvan: multi-core framework for decision diagrams. Int J Softw Tools Technol Transfer.

[48] Saeed MT, Ahmad J, Kanwal S, Holowatyj AN, Sheikh IA, Paracha RZ, Shafi A, Siddiqa A, Bibi Z, Khan M (2016). Formal modeling and analysis of the hexosamine biosynthetic pathway: role of o-linked n-acetylglucosamine transferase in oncogenesis and cancer progression. PeerJ.

[49] Mbodj A, Junion G, Brun C, Furlong EE, Thieffry D (2013). Logical modelling of drosophila signalling pathways. Mol BioSyst.

[50] Fardini Y, Dehennaut V, Lefebvre T, Issad T (2013). O-glcnacylation: a new cancer hallmark?. Front Endocrinol.

[51] Ying H, Kimmelman AC, Lyssiotis CA, Hua S, Chu GC, Fletcher-Sananikone E, Locasale JW, Son J, Zhang H, Coloff JL, Yan H, Wang W, Chen S, Viale A, Zheng H, Paik J-h, Lim C, Guimaraes AR, Martin ES, Chang J, Hezel AF, Perry SR, Hu J, Gan B, Xiao Y, Asara JM, Weissleder R, Wang YA, Chin L, Cantley LC, DePinho RA (2012). Oncogenic kras maintains pancreatic tumors through regulation of anabolic glucose metabolism. Cell.

[52] Gu Y, Mi W, Ge Y, Liu H, Fan Q, Han C, Yang J, Han F, Lu X, Yu W (2010). Glcnacylation plays an essential role in breast cancer metastasis. Cancer Res.

[53] Mi W, Gu Y, Han C, Liu H, Fan Q, Zhang X, Cong Q, Yu W (2011). O-glcnacylation is a novel regulator of lung and colon cancer malignancy. Biochim Biophys Acta (BBA) - Mol Basis Dis.

[54] Zhu Q, Zhou L, Yang Z, Lai M, Xie H, Wu L, Xing C, Zhang F, Zheng S (2012). O-glcnacylation plays a role in tumor recurrence of hepatocellular carcinoma following liver transplantation. Med Oncol.

[55] Rozanski W, Krzeslak A, Forma E, Brys M, Blewniewski M, Wozniak P, Lipinski M (2012). Prediction of bladder cancer based on urinary content of mgea5 and ogt mrna level. Clin Lab.

[56] Krześlak A, Wójcik-Krowiranda K, Forma E, Bieńkiewicz A, Bryś M (2012). Expression of genes encoding for enzymes associated with o-glcnacylation in endometrial carcinomas: clinicopathologic correlations. Ginekol Pol.

[57] Lynch TP, Ferrer CM, Jackson SR, Shahriari KS, Vosseller K, Reginato MJ (2012). Critical role of o-linked *β*-n-acetylglucosamine transferase in prostate cancer invasion, angiogenesis, and metastasis. J Biol Chem.

[58] Muha V, Müller H-AJ (2013). Functions and mechanisms of fibroblast growth factor (fgf) signalling in drosophila melanogaster. Int J Mol Sci.

[59] Glazer L, Shilo B-Z (1991). The drosophila fgf-r homolog is expressed in the embryonic tracheal system and appears to be required for directed tracheal cell extension.. Gene Dev.

[60] Gonzalez AG, Naldi A, Sanchez L, Thieffry D, Chaouiya C (2006). Ginsim: a software suite for the qualitative modelling, simulation and analysis of regulatory networks. Biosystems.

[61] Shafi A, Manzoor J, Hameed K, Carpenter B, Baker M. Multicore-enabling the mpj express messaging library. In: Proceedings of the 8th International Conference on the Principles and Practice of Programming in Java. ACM: 2010. p. 49–58.

[62] Shannon P, Markiel A, Ozier O, Baliga NS, Wang JT, Ramage D, Amin N, Schwikowski B, Ideker T (2003). Cytoscape: a software environment for integrated models of biomolecular interaction networks. Genome Res.

